# Central retinal vein occlusion in young population: risk factors and outcomes

**DOI:** 10.3389/fmed.2023.1180234

**Published:** 2023-08-04

**Authors:** Jordan Berguig, Youssef Abdelmassih, Georges Azar, Justine Lafolie, Anne Sophie Alonso, Sophie Bonnin, Vivien Vasseur, Martine Mauget-Faysse

**Affiliations:** Rothschild Foundation Hospital, Paris, France

**Keywords:** coagulation, CRVO, high blood pressure, inflammation, ocular hypertension, risk factors, young patient

## Abstract

**Objective:**

To evaluate the risk factors and outcomes of central retinal vein occlusion (CRVO) in young patients (< 40 years).

**Methods:**

In this retrospective monocentric case series, we included young patients (<40 years) with CRVO. Patients’ medical files were analyzed focusing on demographic characteristics, suspected risk factors, ophthalmic work-up, visual acuity (VA), treatment, and outcomes.

**Results:**

A total of 54 eyes of 52 patients were included. Central retinal vein occlusion was considered idiopathic in 13 patients (25%). The main risk factors identified were ocular hypertension (20.4%), inflammation (20.4%), high blood pressure (14.8%), and coagulation abnormality (11.1%). Final VA was lower in patients with high blood pressure and inflammation when compared to patients with no risk factor (*p* = 0.03 and 0.04, respectively). Intravitreal injections were needed in 23 eyes (42.6%) and 19 eyes (35.2%) received panretinal photocoagulation treatment.

**Conclusion:**

Central retinal vein occlusion is frequently associated with risk factors in young patients (75% of patients). In addition to the usual factors found in older patients, such as ocular hypertension and high blood pressure, coagulation abnormality and inflammation were also among the risk factors identified. Young patients with CRVO should be evaluated for the presence of risk factors and patients with high blood pressure or inflammatory findings should be followed carefully since they have a worse outcome.

## Introduction

With an incidence of 0.8 per 1,000 people, central retinal vein occlusion (CRVO) is one of the most prevalent retinal vascular diseases (1, 2). It is manifested by papilloedema, venous tortuosity and retinal hemorrhages (3). Complications include cystoid macular edema (CME), retinal and iris neovascularization, neovascular glaucoma, intravitreal hemorrhage and retinal detachment (3, 4). The traditional risk factors are advanced age, ocular hypertension/glaucoma, cardiovascular diseases with high blood pressure playing a predominant role (5, 6). Thrombophilia and chronic inflammation have also been implicated (7–9). With a mean age ranging from 52 to 78.7 years, CRVO is more common in the elderly, but 10–15% of cases occur in subjects under the age of 40 (10–12). Some consider CRVO in young adult a different entity from that of elderly since risk factors traditionally associated with CRVO are less prevalent in this age group (13). Few studies have reported on the CRVO in young adults rendering our understanding of the disease in this age group limited.

The aim of the study is to assess the ocular and general risk factors involved in the occurrence of CRVO in young subjects (< 40 years), evaluate the treatment and outcomes, and compare the visual acuity (VA) according to risk factors.

## Methods

In this monocentric retrospective case series, we included all patients under the age of 40 who presented to the Rothschild Foundation Hospital (RFH) between January 1, 2015 and December 31, 2020 and were diagnosed with CRVO or hemi-RVO. Patients with branch retinal vein occlusion or who lack data at presentation or did not undergo cardiovascular workup or who were lost to follow-up were excluded. This study was approved by the RFH institutional review board -IRB 0012801- under study number CE_20210126_12_MMT and adhered to the principles of the Declaration of Helsinki.

Patient medical records were collected. The systemic and ocular comorbidities collected in this study were selected on the basis of risk factors suspected, in literature, to be associated with the development of CRVO in young subjects. The following criteria were systematically recorded: patient’s age, clinical presentation, laterality as well as medical and ophthalmological history including diabetes, high blood pressure, and ocular hypertension/glaucoma. Since the beginning of 2020, patients were asked about COVID symptoms and positive PCR results.

At initial presentation and at each follow-up, all patients underwent a complete ophthalmological examination including VA, measurement of intraocular pressure (IOP) by Goldmann applanation tonometry (GAT), slit lamp examination and assessment for rubeosis iridis, fundus examination, fundus photos, and spectral-domain (SD) OCT B-scans. OCT-scans were acquired using Heidelberg Spectralis, Heidelberg, Germany. Fundus photos were obtained using Optos widefield imaging system, Nikon, Scotland. Fluorescein angiography (FA) was performed using Optos widefield imaging to distinguish between ischemic and non-ischemic CRVO and to look for ocular inflammation such as retinal vasculitis and papillitis. Ischemic CRVO was defined as an area of nonperfusion of more than 75 disc area (14).

Once the diagnosis was made, the patients were all referred to a specialist for a cardiovascular assessment. In addition, a blood sample was taken to look for coagulation disorders [including prothrombin time (PT), activated partial thromboplastin time (aPTT), antithrombin, protein C, protein S, Factor V Leiden mutation, Prothrombin G20210A mutation, anti-phospholipid antibodies, and circulating type antibodies for lupus] and biologic inflammatory syndrome [erythrocyte sedimentation rate (ESR), C-reactive protein (CRP), serum protein electrophoresis, and fibrinogen]. Visual acuity analysis was converted from Snellen chart to logMAR. Patients with ischemic CRVO and/or rubeosis iridis underwent a panretinal photocoagulation (PRP). Patients with cystoid macular edema received intravitreal injections of antiVEGF or corticosteroids. The treatment choice was left to the discretion of the treating physician.

Statistical analysis was done using the IBM SPSS software (version 22.0, Chicago, IL, United States). Descriptive statistics were reported as mean ± standard deviation for continuous variables and as percentage for categorical variables. Mann–Whitney *U* test was used to compare non-parametric continuous variables between etiology groups. A value of p of less than 0.05 was considered statistically significant.

## Results

### Baseline characteristics

**Figure 1:**
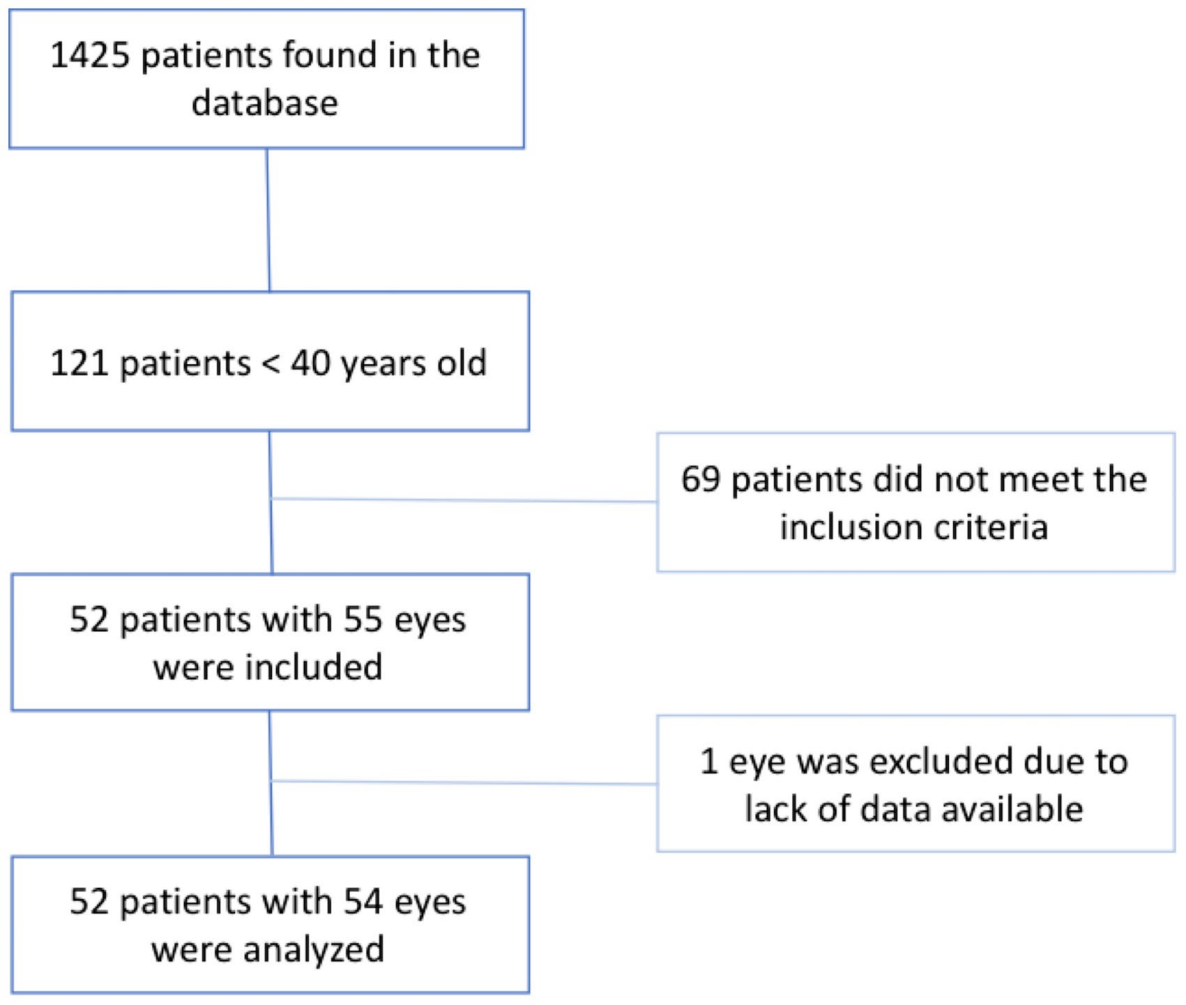
Study flow chart.

A total of 1,425 patients were diagnosed with CRVO of which 121 patients (8.5%) were under the age of 40 at presentation. Out of the 121 patients, 52 (55 eyes) met the inclusion criteria. Finally, 1 eye that had CRVO in the past was excluded due to lack data at baseline, however the second eye of this same patient was included in the study and the patient was considered to have a bilateral CRVO. The results are presented in the study flow chart (Figure 1).

A total of 52 patients (54 eyes) with a mean age at diagnosis of 30.8 ± 7.6 years (range: 8–39 years) and male predominance (28 patients; 53.8%) were included. The disease was bilateral in 3 patients (5.8%) and only 2 patients (3.8%) had hemi-RVO. At presentation, mean IOP was 17.7 ± 7.4 mmHg, and mean VA was 0.65 ± 0.84 logMAR. The baseline characteristics of the patients included in this study are summarized in Table 1. Of the 22 eyes (40%) with retinal ischemia on FA, 17 were considered to have an ischemic CRVO. Intravitreal injections (anti-VEGF or corticosteroids) were needed in 23 eyes (42.6%) and 19 eyes (35.2%) underwent PRP. Four eyes (7.4%) developed rubeosis iridis during follow-up and all had at least one risk factor. The number of patients needing intravitreal injections was comparable between ischemic and non-ischemic CRVO (52.9 vs. 55.9%, respectively, *p* = 0.84).

**Table 1:** Baseline characteristics of included patients.

*Age in years*	
Mean (SD)	30.8 (7.6)
Range	8–39
*Sex, number of patients*	
Female	24 (46.2%)
Male	28 (53.8%)
*Laterality, number of patients*	
Right	27 (51.9%)
Left	22 (42.3%)
Bilateral	3 (5.8%)
*Type, number of eyes*	
HemiRVO	2 (3.7%)
CRVO	52 (96.3%)
*VA at presentation in logMAR*	
Mean (SD)	0.65 (0.84)
*IOP at presentation in mmHg*	
Mean (SD)	17.7 (7.4)
*Follow-up in months*	
Mean (SD)	19.6 (18.8)

CRVO: central retinal vein occlusion, IOP: intraocular pressure, SD: standard deviation, VA: visual acuity.

### Risk factors

Up to 75% of patients (39 patients) had at least 1 risk factor with 6 patients having multiple associated risk factors. The four most common were: ocular hypertension/glaucoma (11 patients: 21.1%), high inflammatory markers/ocular inflammation (11 patients: 21.1%), high blood pressure (8 patients: 15.4%) and coagulation disorders (6 patients: 11.5%). Three patients (5.8%) had received a kidney transplantation of which 2 had a bilateral CRVO and of these 2 patients 1 had a history of uncontrolled high blood pressure. Two patients (3.6%) had a history of optic nerve tumor consisting of optic nerve meningioma for one and metastatic infiltration of the optic nerve secondary to breast cancer for the other. Two patients (3.6%) developed CRVO following blunt ocular trauma, of which one had severe hyphema complicated by ocular hypertension requiring anterior chamber washout. Finally, one patient who developed a mixed venous and arterial occlusion reported nasal cocaine intake with no other risk factors. None of the patients had Covid-19 infection prior, during or following the development of CRVO. All of the risk factors identified in this study are presented in Table 2. At least one risk factor was found in 94.1% of patients with ischemic CRVO (16/17) compared to 68.8% of patients without ischemic CRVO (24 out of 35; *p* = 0.04).

**Table 2:** Central retinal vein occlusion risk factors.

*Risk factor, number of patients*	
None	13 (25%)
Unique	33 (63.5%)
Multiple	6 (11.5%)
*Etiology, number of eyes (%)*	
Ocular hypertension / Glaucoma	11 (21.1%)
Inflammation	11 (21.1%)
High blood pressure	8 (15.4%)
Coagulation abnormality	6 (11.5%)
Kidney transplant	3 (5.8%)
Optic nerve tumor	2 (3,8%)
Eye injury	2 (3.8%)
Drug intake	1 (1.9%)

#### Ocular hypertension and glaucoma

Eleven patients (21.1%) had ocular hypertension or were followed for glaucoma at the time of diagnosis. Mean IOP at presentation in this group was 28.3 ± 8.8 (range: 13–46 mmHg). Among these patients, two were followed and treated for glaucoma.

#### Inflammation

An inflammatory origin was considered if the patient presented with an elevation of one of the biological inflammatory markers (ESR, CRP, fibrinogen or abnormal plasma protein electrophoresis) and/or vasculitis or papillitis diagnosed on FA and detected in 11 patients (21.1%). Among these patients, 5 (45.5%) had an abnormal ESR, CRP and/or fibrinogen level, 2 (18.2%) had an abnormal plasma protein electrophoresis, and 6 patients (54.5%) presented inflammatory anomalies on FA. One patient was diagnosed with neuromeningeal tuberculosis in the follow-up period.

#### Coagulation disorders

Six patients (11.5%) had a coagulation disorder: 4 had an isolated hyperhomocysteinemia, 1 had circulating anticoagulant antibody and hyperhomocysteinemia, and 1 had a Prothrombin G20210A mutation.

### Visual acuity

Mean VA for all etiologies improved from 0.68 ± 0.83 logMAR at presentation to 0.49 (±0.79) logMAR at last follow-up (*p* = 0.03). When no etiology was found, VA improved from 0.35 ± 0.52 logMAR at presentation to 0.15 ± 0.23 logMAR at last follow-up. Patients with ocular hypertension had a worse VA at presentation than patients with no etiology (1.03 ± 0.87 vs. 0.35 ± 0.52 logMAR; *p* = 0.02). Visual acuity at last follow-up in patients with ocular inflammation and patients with high blood pressure was worse when compared to patients with no etiology (*p* = 0.03 and 0.04, respectively). The results are shown in Table 3. Patients with multiples risk factors and those with no risk factors had similar initial BCVA (0.35 ± 0.52 vs. 0.95 ± 0.79; *p* = 0.14) and final BCVA (0.15 ± 0.23 vs. 0.75 ± 0.86; *p* = 0.15). However, patients with ischemic CRVO had similar initial BCVA (0.88 ± 0.93 vs. 0.52 ± 0.71; *p* = 0.18) but a worse final BCVA (0.67 ± 0.83 vs. 0.40 ± 0.80; *p* = 0.03) when compared with non-ischemic CRVO.

**Table 3:** Evolution of visual acuity in logMAR according to the most frequent etiologies.

Etiologies	All patients	None	Ocular hypertension/glaucoma	Inflammation	High blood pressure	Coagulation abnormality
Number of eyes (%)	54 (100)	13 (24.5)	11 (20.8)	11 (20.8)	8 (15.1)	6 (11.3)
Mean VA (±SD) inclusion (value of p)	0.65 (±0.40)	0.35 (±0.52)	1.03 (±0.87) (*p* = 0.02)	0.30 (±0.41) (*p* = 0.80)	0.76 (±0.57) (*p* = 0.11)	0.58 (±0.87) (*p* = 0.47)
Mean VA (±SD) last follow up (value of *p*)	0.49 (±0.80)	0.15 (±0.23)	0.34 (±0.58) (*p* = 0.29)	0.80 (±0.96) (*p* = 0.03)	0.65 (±0.76) (p = 0.04)	0.13 (±0.28) (*p* = 0.87)
Intravitreal injection (%) (value of *p*)	23 (42.6%)	8 (61.5%)	4 (36.4%) (*p* = 0.26)	3 (27.3%) (*p* = 0.12)	4 (50%) (*p* = 0.67)	3 (50%) (*p* = 1.0)
Laser treatment (%) (value of *p*)	19 (35.2%)	5 (38.5%)	5 (45.5%) (*p* = 1.0)	4 (36.4%) (*p* = 1.0)	1 (12.5%) (*p* = 0.36)	1 (16.7%) (*p* = 0.60)

Visual acuities, intravitreal injections and laser treatments of each etiology were compared to those with no risk factor. SD: standard deviation, VA: visual acuity.

## Discussion

In this retrospective case series, we reviewed the clinical characteristics, treatment and outcomes of young patients (< 40 years) with CRVO. This age group represented only 8.5% of the total number of CRVO cases. Similar results were observed in earlier publications with 8% reported by Vannas and Raitta (15) and 15% by Walters and Spalton (8). A more recent large study found that only 3.7% of CRVO occurred in the population of 45 years or less and this percentage significantly increased to 27.3% when the age cutoff was increased to 55 (16). Eah et al. (17) found similar results with 26.2% of CRVO occurring in the population of 50 years or less emphasizing the fact that the incidence of CRVO increases significantly with age. In fact, only 2 patients in our cohort were under the age of 18: one 8-year old patient with a posttraumatic ocular hypertension and hyphema, and one 10-year old patient with an optic nerve tumor. We decided to analyze them as one group, similar to other studies, since the number of children and adolescent with CRVO is limited (17, 18).

Central retinal vein occlusion is predominantly a unilateral disease; in fact, only 5.8% of patients in our cohort had a bilateral involvement. Furthermore, CRVO more frequently affected the right eye (51.9 vs. 42.3%) with a slight male predominance (53.8%) in our cohort. Li et al. (16), who reviewed all the patients presenting with CRVO regardless of age, reported a left-eye onset preference and a higher prevalence of bilateral presentation (9.3%). This might indicate a change in eye onset preference (right to left) and an increase in the risk of the disease becoming bilateral occurring with elderly population. However, gender predominance does not seem exist in young population as it varies between studies with slight difference between genders (16, 17).

A recent study reviewed the etiology of all CRVO cases in young patients and almost always suspected at least one etiology (19). However in our cohort, the search for an etiology remained unsuccessful in 25% of cases. This high frequency might be due to the lack of a standardized list of investigations and the possible role of the COVID-19 virus as the screening using a PCR was not carried out systematically. In fact, recent studies have shown that COVID-19 infection could increase the risk of developing retinal vascular disease due to systemic inflammation and increased risk of thrombosis (20, 21). In the remaining 75% of cases in our cohort, at least one etiology was suspected, with 11.5% having a combination of several risk factors, highlighting the fact that the cause of CRVO can be multifactorial. The most frequent etiologies in order of frequency were: ocular hypertension/glaucoma (21.1%), inflammation (21.1%), high blood pressure (15.4%), and coagulation anomalies (11.5%). Systemic vascular risk factors are the most frequent factors associated with the development of CRVO in elderly; however, this was not observed in young population (22, 23).

### Ocular hypertension/glaucoma

Ocular hypertension and glaucoma are well-known risk factors for CRVO in the general population (24) in fact, a meta-analysis estimated that glaucoma increased by a factor of 4 the risk of developing CRVO (6). We found it to be the most frequent risk factor similarly to Chen et al. who found open-angle glaucoma to be the first risk factor of OVR with no gender predilection (23). The increase in IOP may result in the compression of central retinal vein occlusion against the lamina cribrosa causing a turbulent flow, a higher risk of thrombus formation, and thus CRVO (23).

### Inflammation

Inflammation is a documented cause of CRVO in young population (25). In our study, inflammatory markers were as frequent as OHT. In addition, unlike other risk factors, visual acuity at last follow-up in these patients was worse than patients with no apparent risk factor. Similarly, Chen et al. (23) found a strong and significant association between retinal vasculitis and CRVO. High level of endothelin-1 was found to be a potential risk factor for the development of all types RVO (26). More recent studies have reported CRVO to be a complication COVID-19 infection, potentially linked to systemic inflammation and the increased risk of thrombosis (20, 21). When CRVO occurs in a young patient, it is important to evaluate for inflammatory markers by assessing biologic inflammatory indicators (such as ESR, CRP, serum protein electrophoresis, and fibrinogen) and performing FA to assess for retinal vasculitis. Yoshizawa et al. (9) reported that treating cases of idiopathic inflammation with systemic corticosteroid could improve the final visual acuity. However, tuberculosis and toxoplasmosis should be excluded prior to starting patients on systemic corticosteroids.

### Hypertension and cardiovascular diseases

Hypertension was found to be one of the main risk factors associated with CRVO in elderly increasing the risk of developing CRVO around 3.5 times (22). This is not surprising considering that high blood pressure promotes the process of atherosclerosis (27). However, hypertension was less prevalent among younger patients with CRVO in fact it represents the 3rd etiology in our cohort. None of our patients had a history of diabetes. Eah et al. (17) reported that older patients had notably higher prevalence of hypertension when compared to younger population. Chen et al. (23) found using regression analysis that hypertension was not as significant risk factor for CRVO in young adults. Hypertension becomes more prevalent with age and this may be due to the cumulative vascular change secondary atherosclerosis which becomes more clinically significant with longstanding hypertension.

Since the usual risk factors for CRVO are less frequent in younger population and that CRVO in more prevalent in elderly other less usual risk factors should be evaluated when CRVO develops in a young patients. These risk factors include coagulation anomalies (28), hyperviscosity secondary to vascular tumors (29), tumor of the optic nerve secondary to the compression of the central retinal vein, eye trauma (30), complications of end-stage kidney disease or transplant (31), and drugs intake. Zhang et al. (19) proposed an extensive workup for CRVO in young population.

### Outcomes and treatment

Visual acuity significantly improved from 0.65 ± 0.84 to 0.49 ± 0.81 at last follow-up. Laser treatment was needed in 35.2% of eyes and antiVEGF treatment in 42.6%. Despite the big disparity in baseline and last visual acuity between studies, there was always a significant improvement in VA with some studies finding that younger population had a milder course (17, 23, 32). The need for intravitreal injections was similar to Eah et al. (17) (47.8%) but lower than Koh et al. (32) (66%). This difference could be due to difference in age and to different risk factors between populations. Laser treatment was similar to Eah et al. (17) (42.9%). However, it seems that CRVO in the young population is less ischemic and some believe that they may need less laser treatment (17, 19).

The limitations of the study are its retrospective design, and the exclusion of patients that were lost to follow-up or did not undergo the workup which limits the number of included patients. A further limitation may be the selection of patients from a tertiary referral center and who underwent an extensive workup which increases the prevalence of risk factors. It is noteworthy that patients were not tested for COVID-19 which could have been a possible additional risk factor.

## Conclusion

Central retinal vein occlusion is a multifactorial pathology. Despite the fact that the exact cause has yet to be fully elucidated, venous stasis responsible for a thrombus is one of the hypothesis. Although present, traditional risk factors (hypertension and diabetes) for CRVO are less frequent in young population. Ocular hypertension/glaucoma and inflammation are the most frequent risk factors. The prognosis of CRVO varies according to the risk factors. Inflammation and high blood pressure had a worse final BCVA when compared to idiopathic cases. Other less frequent risk factors should be looked for especially coagulation abnormalities. Patients seem to need less intravitreal injections and laser treatment than the older group.

## Data availability statement

The original contributions presented in the study are included in the article/supplementary material, further inquiries can be directed to the corresponding author.

## Ethics statement

The studies involving human participants were reviewed and approved by RFH institutional review board -IRB 0012801- under study number CE_20210126_12_MMT. The patients/participants provided their written informed consent to participate in this study.

## Author contributions

YA, JB, MM-F, and SB substantially contributed to the conception or design of the work. GA, JL, AA, and VV contributed to the acquisition, analysis, or interpretation of data for the work. YA, JB, MM-F contributed to the drafting of the work. GA, JL, ASA, SB, and VV contributed to revising it critically for important intellectual content. JB, YA, GA, JL, AA, SB, VV, and MM-F were responsible for all aspects of the work in ensuring that questions related to the accuracy or integrity of any part of the work are appropriately investigated and resolved. All authors contributed to the article and approved the submitted version.

## Conflict of interest

The authors declare that the research was conducted in the absence of any commercial or financial relationships that could be construed as a potential conflict of interest.

## Publisher’s note

All claims expressed in this article are solely those of the authors and do not necessarily represent those of their affiliated organizations, or those of the publisher, the editors and the reviewers. Any product that may be evaluated in this article, or claim that may be made by its manufacturer, is not guaranteed or endorsed by the publisher.
